# Rhythms in Decoding Do Not Signal Pulsing Information

**DOI:** 10.1111/ejn.70725

**Published:** 2026-10-16

**Authors:** Freek van Ede

**Affiliations:** ^1^ Institute for Brain and Behavior Amsterdam, Department of Experimental and Applied Psychology Vrije Universiteit Amsterdam Amsterdam the Netherlands; ^2^ Departament de Cognició, Desenvolupament i Psicologia de l’Educació, Secció Processos Cognitius, Facultat de Psicologia, Institut de Neurociències Universitat de Barcelona (UB) Barcelona Spain

## Abstract

Emerging studies report that information decoded from brain activity may pulse at specific frequencies. As such inferences take root, I clarify that trial‐level decoding time courses inherit background brain rhythms even when information content is stable. This cautions against conflating rhythms in decoding with rhythms in information content.

Decoding analyses—such as multivariate pattern analyses—offer a powerful approach for tracking information content contained in patterns of neural activity (Haynes and Rees 2006; Kriegeskorte et al. 2006; Stecher et al. 2025; Stokes et al. 2015; Tong and Pratte 2012). They allow studying representational contents over and above the timing and amplitude of neural responses. Without a doubt, such decoding approaches have been a major advance in cognitive neuroscience, offering inferences that more directly speak to the nature of cognitive processes and the information they act upon. However, an emerging development in inferring cognitive processing from decoding outputs calls for scrutiny, in the wake of unwarranted inferences.

Setting the stage for the development in question, a burgeoning series of studies has reported how cognitive processes may be inherently rhythmic (Fiebelkorn and Kastner 2019; Landau and Fries 2012; VanRullen 2016). For example, rhythmic fluctuations in task performance have been noted following the onset of attention cues (Fiebelkorn and Kastner 2019; Landau and Fries 2012) or movements (Tomassini et al. 2015), and complementary studies have linked the phase of ongoing brain oscillations to task performance (Busch et al. 2009; Darrell et al. 2026; Fiebelkorn et al. 2018; Stefanics et al. 2010). Such findings have led to the notion that core cognitive processes may fluctuate rhythmically.

Fueled by such studies, multiple recent studies have started to apply spectral frequency analyses to trial‐level outputs of decoding analyses to look for rhythmic “pulsing” of the informational content that is carried by neural activity patterns (Griffiths 2026; Hetenyi et al. 2025; Siems et al. 2026). So far, claims for such pulsing—in which representations reportedly go “online” and “offline” in cycles—have already been reported for information regarding predicted stimulus identity (Hetenyi et al. 2025), the covert locus of attention during decision making (Siems et al. 2026), or associations retrieved from long‐term memory (Griffiths 2026).

Strikingly, each of these studies converged on information content pulsing at approximately 10 Hz, in the so‐called alpha band (Berger 1929). This is noteworthy because this also happens to be the dominant frequency in the “background brain activity” fed to the classifier in these studies that each relied on noninvasive electrophysiological measurements of human brain activity. Here, I show how this is an inevitable outcome and explain why caution should be exercised before inferring that information content itself fluctuates rhythmically.

Caution is warranted because single‐trial decoding time courses naturally “inherit” background signal properties that do not themselves contain the decoded information. The reason for this is simple—even if also easy to overlook amidst complex analysis pipelines. If the single‐trial activity that is fed into the classification algorithm itself oscillates at a specific frequency (something measurements of electrophysiological brain activity tend to do), then the distance to the classification boundary that separates matching and nonmatching trial classes will also oscillate. In other words, if the trial‐level data fed into the classifier oscillates, then so will the classifier output. Critically, as I illustrate in Figure 1, this will happen even when this true (trial‐average) distance between matching and nonmatching classes—and therefore the true classification boundary—is stable.

**FIGURE 1:**
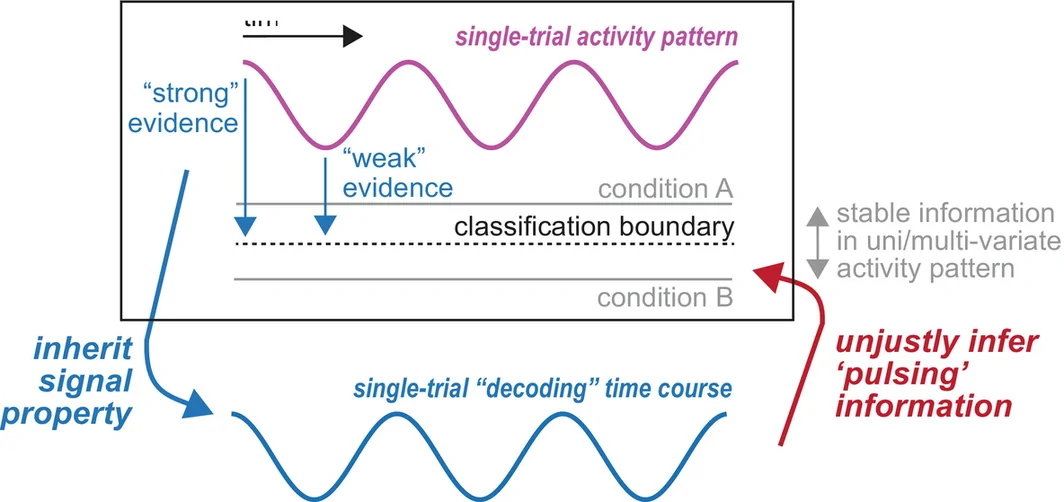
Single‐trial decoding time courses inherit background brain rhythms even when information content is stable.

Thus, trial‐level decoding output will inevitably inherit background brain rhythms (and any other temporal property of the signal fed into the classifier). However, such rhythmic decoding output should not be confused with—nor used to infer—rhythmic pulsing of the decoded “information content.” This is the central takeaway and call for caution of this article.

Though the issue is clear, potential ways around it are less obvious and await clever solutions.

First, I note how the issue is particularly obvious when relying on continuous decoding evidence because such decoding outputs directly preserve fluctuations in the distance from the classification boundary. Yet, the same fundamental issue persists when using discrete accuracy scores coming out of a binary or multiclass classification approach. This is because also discrete classification outputs will oscillate whenever background brain rhythms oscillate around the classification boundary.

Another approach that appears a solution at first sight is to generate a baseline with shuffled surrogate data (such as by randomly permuting condition labels). However, at the core, shuffling does not solve the concern either. While shuffling will remove the true difference between conditions, it will leave the inheritance of trial‐level signal properties unaffected. Indeed, even shuffled data have been reported to have a clear alpha peak in the single‐trial decoding time courses (Hetenyi et al. 2025), calling into question the appropriateness of such shuffling when drawing inferences on frequency‐specific pulsing of decoded information content. Of note, besides this challenge with surrogate data when shuffling condition labels across trials, complementary challenges are faced when creating surrogate data by shuffling data points across time as this can yield surrogate data that does not preserve the aperiodic spectral structure in the data (see Brookshire 2022 for elaborate coverage of this complementary challenge).

But what to infer when the decoding output from the data prior to shuffling shows a larger spectral peak at a specific frequency than the shuffled data? If a spectral peak survives after subtracting the spectrum in the shuffled data, then would this not count as good evidence for pulsing information content? Again, scrutiny and caution are called for. Such a finding may still reflect a true difference that is stable rather than pulsing. This is because, in the presence of a true difference, background brain rhythms will be inherited more veridically. After shuffling, classifier evidence in the trial‐level data relative to Conditions A and B will more often flip sign owing to Conditions A and B having been randomized. This will lead to an inherited rhythm in the decoding output of the shuffled data with more frequent phase reversals, and such a distorted rhythm will have lower amplitude when subjected to standard frequency analysis.

Before the widespread analysis of rhythmicity in decoding outputs takes a firmer hold across electrophysiological studies in cognitive neuroscience, I thus caution for the inferences that may intuitively, but erroneously, follow. I stress that my aim is not to take a theoretical position on whether cognitive processes—and the information content they act on—may or may not pulse rhythmically in the brain. Nor do I question that spectral activity, such as the power in specific frequency bands, can contain decodable information (Stecher et al. 2025). I merely reflect on the emerging practice of subjecting trial‐level decoding time courses to spectral analyses as a form of evidence for rhythmic information processing. I have explained why such evidence is easy to overinterpret and fundamentally unsuitable for demonstrating rhythmic pulsing of information content (until appropriate solutions have been found that overcome the issues outlined above). Trial‐level decoding outputs that peak in specific frequencies may reflect nothing but the background brain rhythms that enter the classification algorithm, according to a “rhythm in, rhythm out” principle.

## Author Contributions

**Freek van Ede:** conceptualization, investigation, funding acquisition, writing – original draft, visualization.

## Conflicts of Interest

The author declares no conflicts of interest.

## Data Availability

Data sharing is not applicable to this article as no datasets were generated or analyzed during the current study.
